# A dynamic view of the proteomic landscape during differentiation of ReNcell VM cells, an immortalized human neural progenitor line

**DOI:** 10.1038/sdata.2019.16

**Published:** 2019-02-19

**Authors:** Yuyu Song, Kartik Subramanian, Matthew J. Berberich, Steven Rodriguez, Isabel J. Latorre, Catherine M. Luria, Robert Everley, Mark W. Albers, Timothy J. Mitchison, Peter K. Sorger

**Affiliations:** 1 Laboratory of Systems Pharmacology, Program in Therapeutic Science, Harvard Medical School, Boston, MA 02115, USA; 2 Department of Systems Biology, Harvard Medical School, Boston, Massachusetts 02115, USA; 3 Department of Neurology, Massachusetts General Hospital, Boston, Massachusetts 02114, USA; 4 Department of Cell Biology, Harvard Medical School, Boston, Massachusetts 02115, USA

**Keywords:** Proteomics, Mechanism of action, Proteome informatics, Differentiation, Neural progenitors

## Abstract

The immortalized human ReNcell VM cell line represents a reproducible and easy-to-propagate cell culture system for studying the differentiation of neural progenitors. To better characterize the starting line and its subsequent differentiation, we assessed protein and phospho-protein levels and cell morphology over a 15-day period during which ReNcell progenitors differentiated into neurons, astrocytes and oligodendrocytes. Five of the resulting datasets measured protein levels or states of phosphorylation based on tandem-mass-tag (TMT) mass spectrometry and four datasets characterized cellular phenotypes using high-content microscopy. Proteomic analysis revealed reproducible changes in pathways responsible for cytoskeletal rearrangement, cell phase transitions, neuronal migration, glial differentiation, neurotrophic signalling and extracellular matrix regulation. Proteomic and imaging data revealed accelerated differentiation in cells treated with the poly-selective CDK and GSK3 inhibitor kenpaullone or the HMG-CoA reductase inhibitor mevastatin, both of which have previously been reported to promote neural differentiation. These data provide in-depth information on the ReNcell progenitor state and on neural differentiation in the presence and absence of drugs, setting the stage for functional studies.

## Background & Summary

Better understanding of networks mediating differentiation of neural stem cells into functional neurons and glia holds promise for treating neurodevelopmental disorders^1^, traumatic brain injury and neurodegenerative diseases^2^. Efforts are underway to catalogue genome-wide RNA expression in developing mammalian brains^3–5^, but detailed characterization of signalling still depends on model systems in which differentiation can be reproducibly triggered. One such system is the ReNcell VM immortalized neural progenitor (RRID: CVCL_E921), which was derived from the ventral mesencephalic region of the developing human brain and can be induced to differentiate by growth factor withdrawal. Differentiation generates a mixture of neurons and glia able to form functional electrophysiological circuits in either 2D or 3D. These circuits more closely resemble the human nervous system than do primary neuronal monocultures^6,7^. ReNcell VM cells have therefore become a valuable tool for identifying neurodevelopmental pathways, investigating neuronal function^8,9^ and probing neurodegeneration pathways including those involving the Aβ and tau pathologies observed in Alzheimer’s Disease^7^.

**Figure 1:**
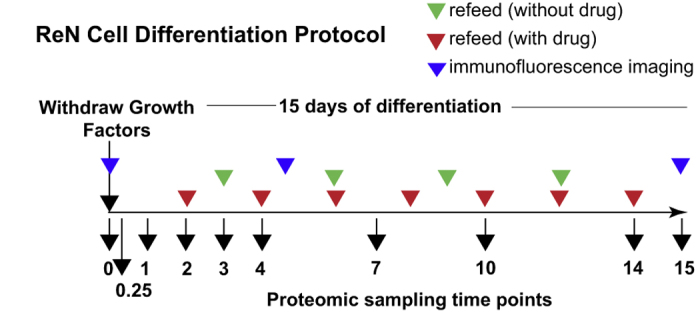
Workflow for investigation of ReNcell VM differentiation.

**Figure 2:**
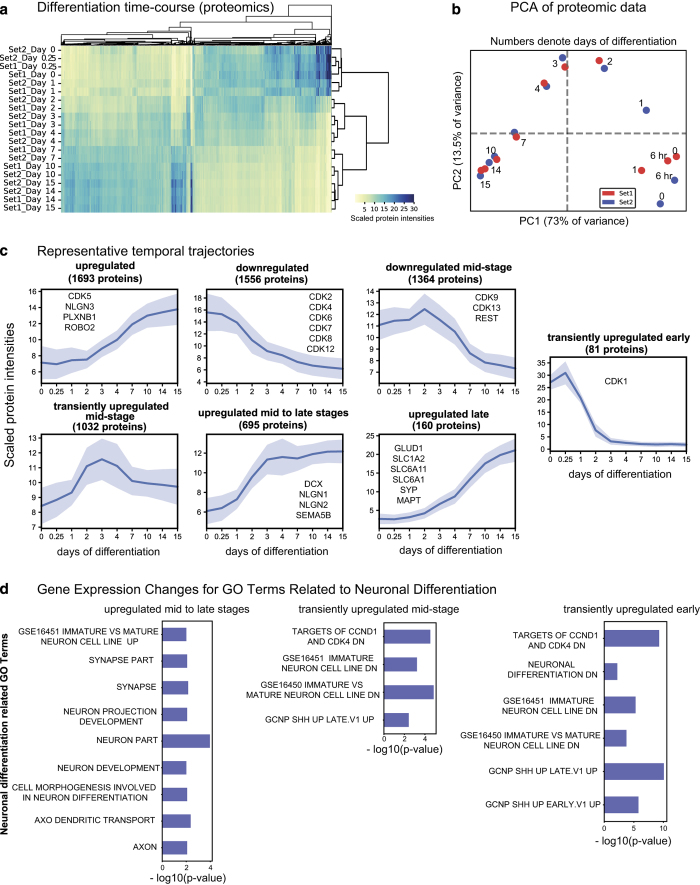
Changes in total proteome as measured by shotgun proteomics. (**a**) Hierarchical clustering of relative protein abundance measured across 15 days (*n* = 2). (**b**) PCA was performed on proteins in common between data set 1 and 2 (representing 7568 proteins). (**c**) k-means clustering was used to identify distinct temporal patterns. The groups are labelled by the trend over time with the numbers of proteins in each group shown in brackets; some representative proteins are also listed. Each plot shows the relative change in protein abundances for one group. (**d**) Enrichment analyses using a custom neuronal gene set library was performed on each cluster. Significantly enriched terms (p-value<0.05) for clusters named ‘upregulated’, ‘downregulated’ and ‘transiently upregulated early’ are shown.

**Figure 3:**
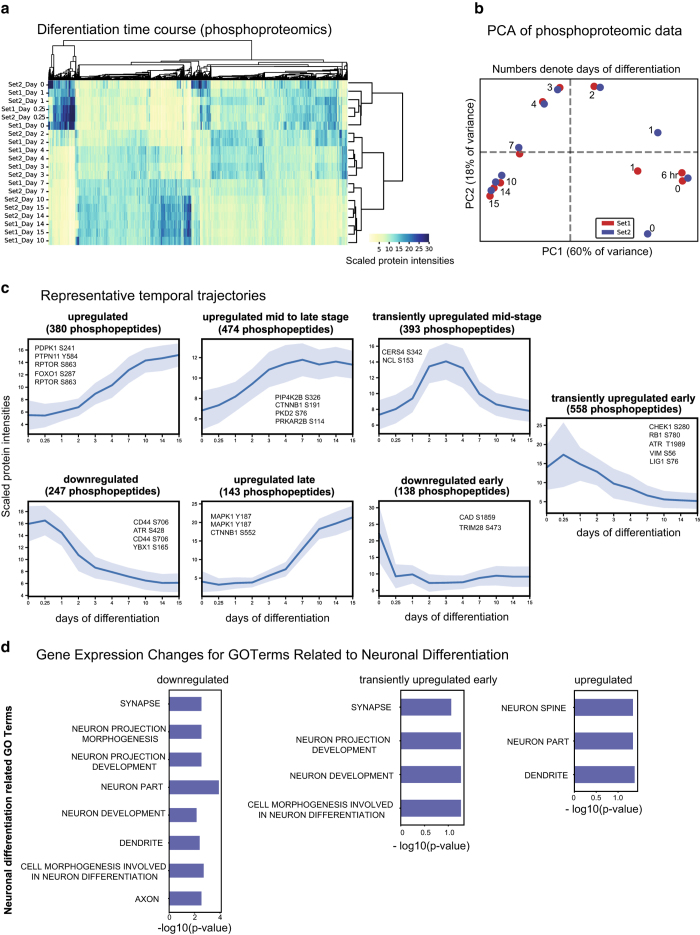
Changes in the phosphoproteome as measured by shotgun proteomics. (**a**) Hierarchical clustering of relative phosphoprotein abundance measured across 15 days (*n* = 2). (**b**) PCA was performed on phosphoproteins in common between Set 1 and Set 2 (2767 phosphoproteins). (**c**) k-means clustering was used to identify distinct temporal patterns. The groups are labelled by the trend over time with the numbers of phosphopeptides in each group shown in brackets; some representative phosphopeptides are also listed. Each plot shows the relative change in phosphopeptide abundances for one group. (**d**) Enrichment analyses using a custom neuronal gene set library was performed on each cluster. Significantly enriched terms (p-value < 0.05) for clusters named ‘upregulated’, ‘upregulated mid to late stage’ and ‘upregulated late’ are shown.

The process of neuronal differentiation has been studied using proteomic methods^8–12^, but full time course data on changes in protein abundance and modification state (phosphorylation in particular) are not currently available for ReNcell VM cells or similar culture systems derived from human neural stem cells. Here, we describe a study in which proteomics was coupled to live and fixed cell imaging to systematically compare changes in ReNcell VM composition and cell state during a 15-day differentiation process (Fig. 1) (syn11701218, Data Citation 1). Liquid chromatography mass spectrometry (LC-MS) using 10-plex tandem mass tag (TMT) labelling, combined with phosphopeptide enrichment by titanium dioxide (TiO_2_)^13^, allowed deep quantification of proteome dynamics across 8,876 proteins and 2,745 phospho-proteins (comprising ~6,863 sites of phosphorylation) (Figs 2, 3) (syn11701290 and syn11701291, Data Citation 1).

**Figure 4:**
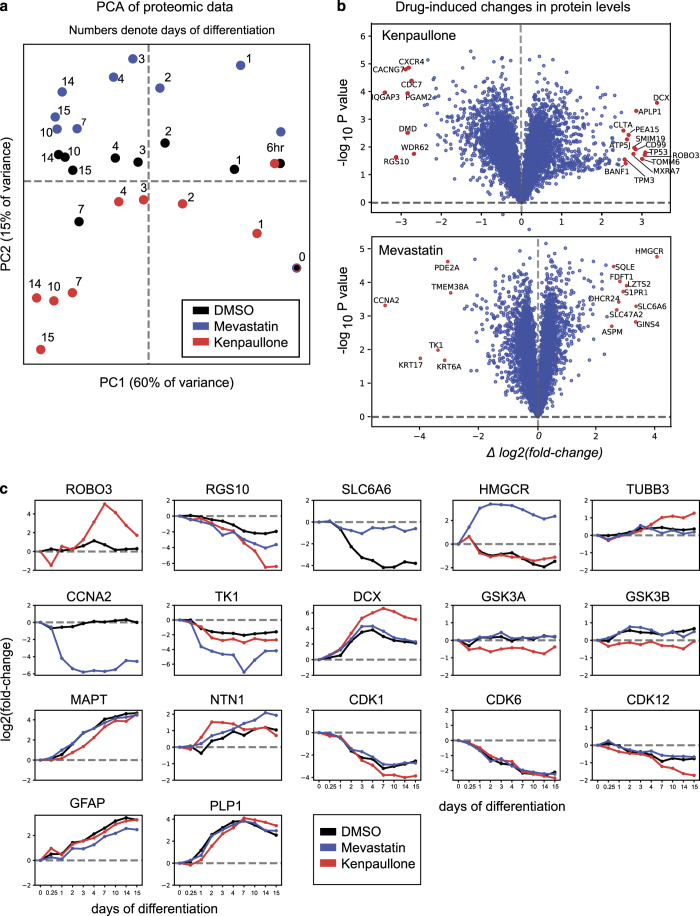
Changes in total proteomes across 15 days after treatment with either DMSO, kenpaullone, or mevastatin. (**a**) Relative protein abundances for DMSO, kenpaullone or mevastatin-treated cells were normalized to their respective Day 0 values. PCA of the normalized datasets shows the position of each sample on PC space. (**b**) Volcano plot for kenpaullone (left panel) and mevastatin (right panel) relative to DMSO. The x-coordinate of each point is the mean delta between the log2(fold-change) of a given protein under treatment (kenpaullone or mevastatin) and DMSO across days 7 to 15. The y-coordinate is the –log10(p-value) computed using single sample t-test on the delta log2(fold-change) values across days 7 to 15. Points marked in red are proteins for which the absolute mean delta log2(fold-change) value is above 2.5 and the p-value is below 0.05. (**c**) The normalized protein abundance values in log2 space plotted from day 0 to 15 for neuronal markers of interest. Black lines represent data from cells treated with DMSO, red with kenpaullone and blue with mevastatin.

Neural differentiation was first studied under standard conditions of growth factor withdrawal. The resulting total and phosphoproteomic data were consistent across biological repeats as indicated by hierarchical clustering (repeats clustered together; Figs 2a and 3a). Principal Component Analyses (PCA) showed a clear progression with time and indicated three distinct stages of differentiation *i.e.* early (days 0 to 1), mid (days 2 to 4) and late (days 7 to 15) (Figs 2b and 3b). Significant changes were observed in the levels and phosphorylation states of proteins involved in stem cell proliferation, cell cycle, apoptosis, neuronal cytoskeleton, astrocytic differentiation, axonal guidance, neurotrophic pathways, extracellular matrix signalling and synaptogenesis (Figs 2c,d and 3c,d). ReNcells were also differentiated in the presence of kenpaullone, a poly-selective cyclin dependent kinase (CDK)/ Glycogen synthase kinase 3(GSK3) inhibitor and mevastatin, an inhibitor of 3-hydroxy-3-methylglutaryl coenzyme A (HMG-CoA) reductase (syn11701292, syn11701293, syn11701294, Data Citation 1). Both drugs have been reported to promote neurite outgrowth in ReNcells^14^. PCA (Fig. 4a) and volcano plots (Fig. 4b) of data collected from drug-treated cells suggest that the molecular changes induced by these drugs are different though they both promote neuronal differentiation. Kenpaullone and mevastatin cause temporal changes in some molecular markers of neural differentiation as well as altering levels of selective protein expressions observed in the absence of drugs (e.g. Fig 4b,c).

**Figure 5:**
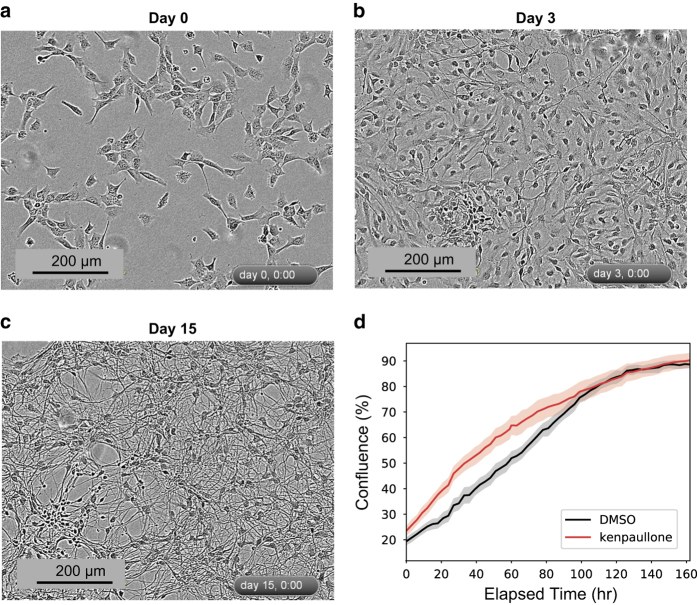
Consistent neural differentiation across independent passages of ReNcell VM cells. (**a**) Live imaging of neural differentiation at 0 day, (**b**) 5 days and (**c**) 15 days. (**d**) Confluency, defined as the total area of neural processes and cell bodies per well × 100, is plotted as a function of time for DMSO control- and kenpaullone-treated cells across 7 days (*n = 4*).

**Figure 6:**
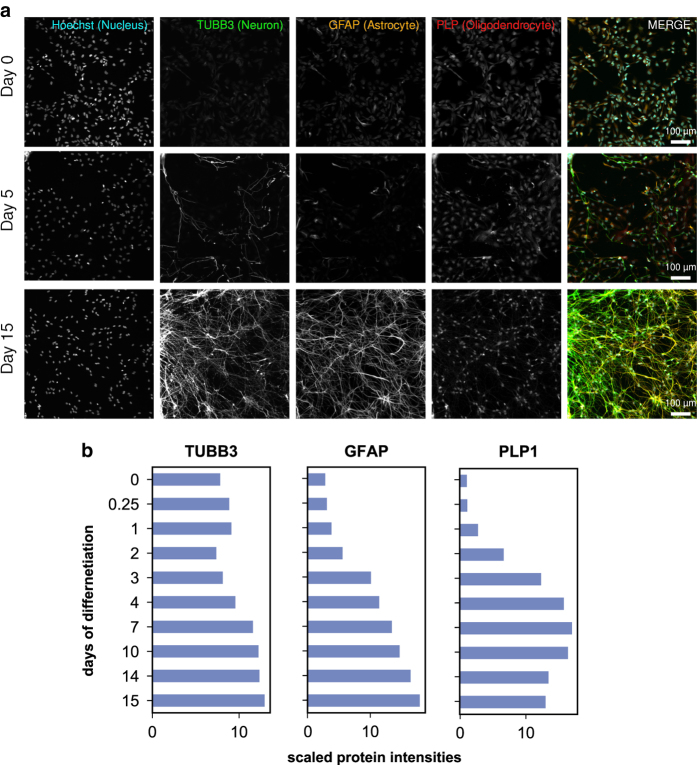
Validation of neuronal/glial differentiation by immunofluorescent imaging (IF). (**a**) ReNcell VM cells differentiate into neurons expressing TUBB3, astrocytes expressing GFAP, and oligodendrocytes expressing PLP1. (**b**) Bar charts show protein levels quantified by total proteomics for comparison.

**Figure 7:**
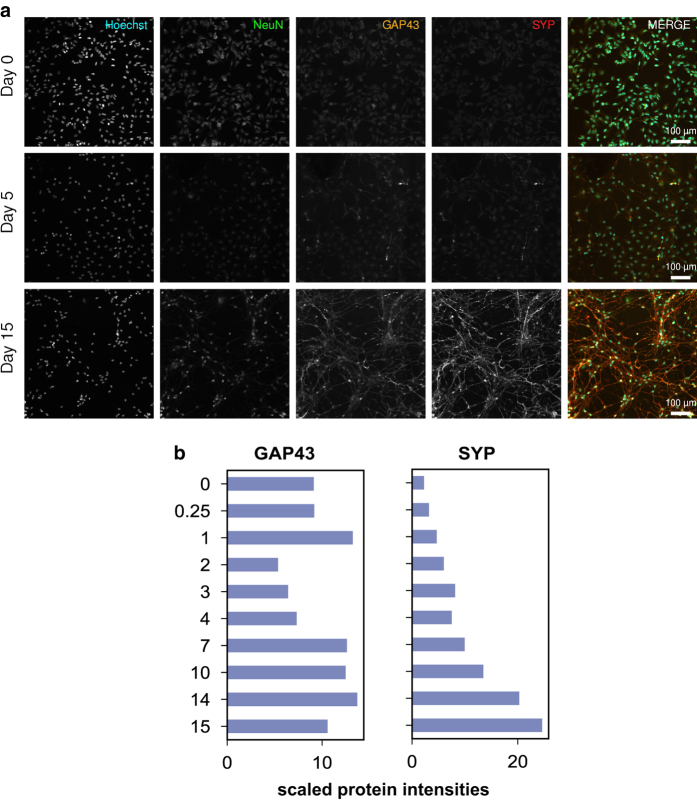
Validation of synaptic development by IF. (**a**) ReN neurons, labelled with NeuN, express increasing levels of GAP43 and Synaptophysin (SYP) during differentiation, indicating growth cone development and synaptogenesis. (**b**) Bar charts show protein levels as quantified by total proteomics for comparison.

**Figure 8:**
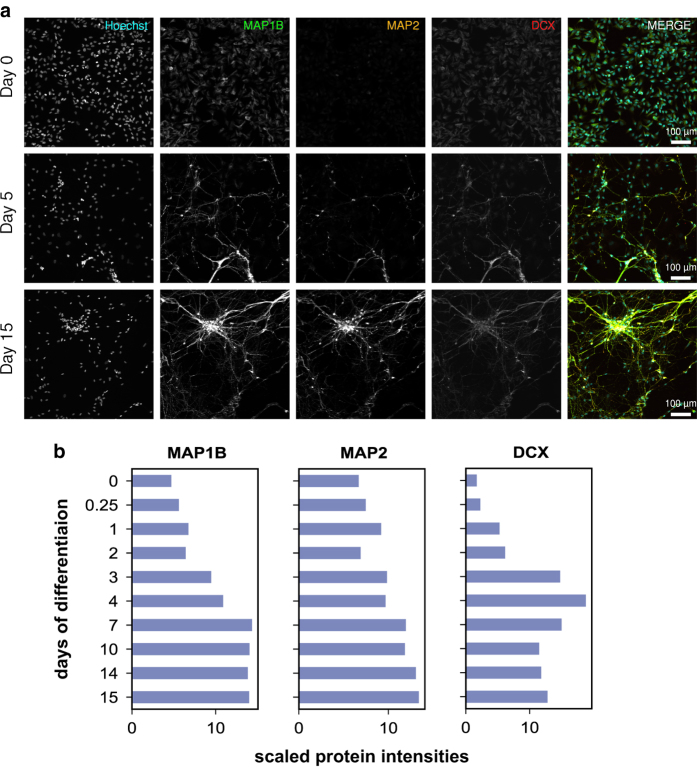
Validation of cytoskeletal maturation by IF. (**a**) Cytoskeletal markers (MAP1B, MAP2 and DCX) increase in differentiated neurons, consistent with fundamental features of neuronal differentiation *in vitro* and *in vivo*. (**b**) Bar charts show protein levels as quantified by total proteomics for comparison.

**Figure 9:**
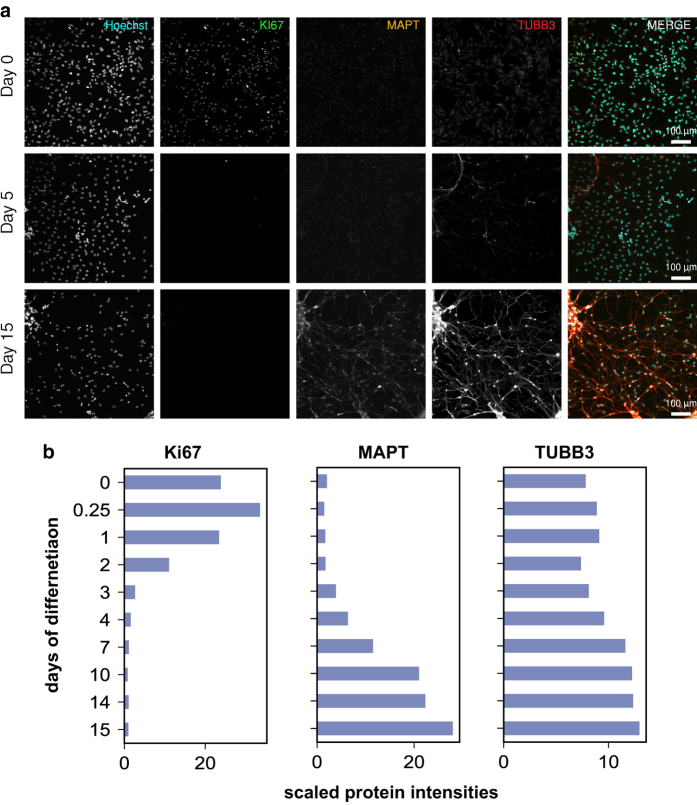
Validation of cell fate transition by IF. (**a**) The cell proliferation marker (Ki67) is inhibited at the start of differentiation and remains low as mature neurons start expressing MAPT (Tau) and Tubb3. (**b**) Bar charts show protein levels as quantified by total proteomics for comparison.

**Figure 10:**
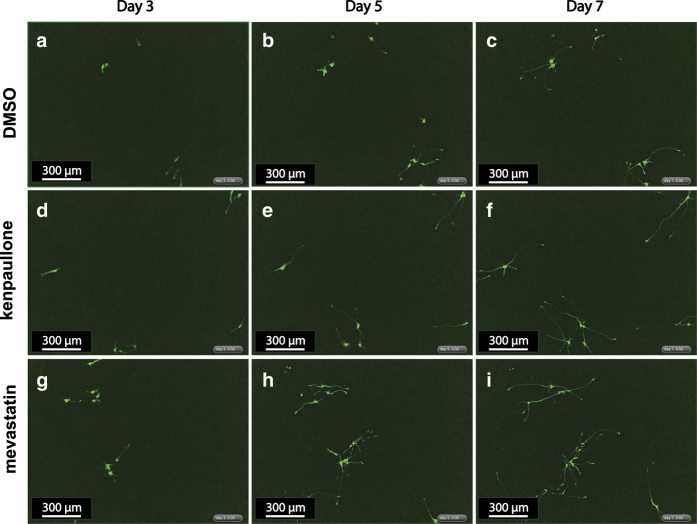
Drug-promoted ReNcell VM-GFP differentiation by live imaging. Both kenpaullone and mevastatin promote neural differentiation as judged by the extent of neurite initiation (**d** and **g**) and neurite elongation (**e** and **h**) as compared with DMSO control (**a** and **b**). All three groups look more comparable on day 7 than earlier time points (**c**,**f**, and **i**).

Live and fixed cell imaging revealed dramatic changes in cell morphology over the course of ReNcell VM differentiation and demonstrated the presence of neurons and glia in the final culture (Figs 5–9) (syn11691420, Data Citation 1). Changes in morphology were highly reproducible across biological replicates (Fig. 5) (syn11725079, syn11701318, Data Citation 1). Kenpaullone and mevastatin were observed to promote neurite extension and increase neurite length, particularly during the first few days after growth factor withdrawal (Fig. 10) (syn11725205, Data Citation 1). These data substantially extend previous information on the differentiation of ReNcell VM cultures and set the stage for detailed functional studies^15^.

## Methods

### Culture Conditions

The human ReNcell VM Immortalized cell line (RRID:CVCL_E921; https://web.expasy.org/cellosaurus/CVCL_E921) was obtained from EMD Millipore (Catalog Number SCC008) and tested for mycoplasma contamination and self-renewal when grown in ReNcell NSC Maintenance Medium (Millipore, Catalog Number SCM005) supplemented with basic fibroblast growth factor (bFGF, Stemgent, Catalog Number 03-0002) and epidermal growth factor (EGF, Sigma, Catalog Number E9644); cells were also tested for the ability to undergo multi-lineage differentiation when grown in Maintenance Medium without added growth factors. Cells were cultured for proteomics experiments in Matrigel-coated 15-cm plates (Corning, Catalog Number 353025) and for imaging experiments in Matrigel-coated 96-well optical-bottom plates (ThermoFisher, Catalog Number 165305). Cells were harvested at ten time points following a switch to growth factor-free medium (t = 0, 6 hr or 1, 2, 3, 4, 7, 10, 14 or 15 days). Culture medium was then changed every three days following the manufacturer’s recommended protocol. The ReN-G line^7^ was provided by Dr. Doo Yeon Kim at Mass General Hospital in Boston, MA; it stably expresses GFP from a lenti-viral vector.

### Drug Treatment

Chemical perturbation studies were performed using mevastatin at 2 μM final concentration (Tocris, Catalog Number 1526, HMS LINCS ID: 10620-101), or kenpaullone at 2 μM (Sigma, Catalog Number K3888-5MG, HMS LINCS ID: 10619-101) or DMSO as a control. Drugs were tested for purity in-house by LC-MS and NMR. QC results and curated metadata can be found at for http://lincs.hms.harvard.edu/db/sm/10619-101-1/#batchinfo kenpaullone and http://lincs.hms.harvard.edu/db/sm/10620-101-1/#batchinfo for mevastatin. Half of the medium was withdrawn every two days and replaced with fresh drug-containing medium, in keeping with a previous study^14^. Cells were harvested at the same time points as in no-drug studies.

### Mass Spectrometry

#### Sample Collection

Cells grown in Maintenance Medium without growth factors for 0 to 15 days were rinsed once with phosphate-buffered saline (PBS) and then gently scraped from 15 cm dishes in PBS supplemented with protease and phosphatase inhibitors (Halt^TM^ Protease and Phosphatase Inhibitor Single-Use Cocktail, EDTA Free, ThermoFisher, Catalog Number 78441) followed by centrifugation at 300 g for 3 minutes. The supernatant was discarded and pellets were stored at −80 °C.

#### Protein Solubilisation and Digestion

Cell pellets were solubilized for 2 minutes by vortexing in lysis Buffer (2% SDS, 150 mM NaCl, 50 mM Tris pH 7.4) supplemented with Halt^TM^ Protease and Phosphatase Inhibitor Single-Use Cocktail, EDTA Free (ThermoFisher, Catalog Number 78443). Cell lysates were then transferred to QiaShredder Mini Spin Columns (Qiagen, Catalog Number 79656), and processed according to the manufacturer’s protocol. Disulfide reduction was performed by adding dithiothreitol (DTT) to a final concentration of 5 mM and heating to 37 °C for 1 hour, followed by alkylation with iodoacetamide at a final concentration of 15 mM and incubation at room temperature in the dark for 30 minutes. Protein concentration was determined using a Micro BCA^TM^ Protein Assay Kit (ThermoFisher, Catalog Number 23235) following the manufacturer’s protocol. For each time point, an aliquot corresponding to 150 μg of total protein was withdrawn. Detergent was removed by methanol/chloroform protein precipitation as described previously^16^. Precipitates were solubilized in freshly prepared 8 M urea in 20 mM EPPS, pH 8.5 and 60 μg of solubilized total protein from each sample was then used for TMT labelling. Following a 10 min incubation at 37 °C, the urea concentration was diluted with 20 mM EPPS to 4 M final concentration and digestion was performed by overnight incubation at room temperature in the presence of Lys-C protease (Wako, Catalog Number 129-02541) at an enzyme-to-substrate ratio of 1:75. Following further dilution of the sample with 20 mM EPPS to a final urea concentration of 1.6 M, further digestion was performed by incubation at 37 °C for 6 hours with trypsin (Promega, Catalog Number V5113) at an enzyme to substrate ratio of 1:75.

#### Digest Check

The missed cleavage rate was determined by LC-MS/MS. 1 μg of total protein was withdrawn from each time point and combined into a single sample. Only samples with a missed cleavage rate <15% were processed further.

#### TMT Labelling, Ratio Check and HPLC Fractionation

Equal amounts of protein were removed from each sample and labelled using a TMT10plex Mass Tag Labelling Kit (ThermoFisher, Catalog Number 90406) as shown (syn11701215, Data Citation 1). TMT labelling efficiency and ratio checks were measured by LC-MS3 analysis of a combined 10-plex sample after combining equal volumes (about 1 μg) from each time point. Equal amounts of labelled peptide from each time point (as judged from ratio check data) were then combined for subsequent analysis.

Quenching of TMT labelling reactions was performed by adding hydroxylamine to a final concentration of 0.5% (v/v) and incubating samples for 10 minutes at room temperature. Formic acid (FA) was added to a final volume of 2% (v/v) to lower the pH below 3.0 and samples were combined and de-salted using a SepPak tC18 Vac RC Cartridge (50 mg, Waters, Catalog Number WAT054960). HPLC fractionation was performed using an Agilent 1200 Series instrument with a flow rate of 600 μl/minute over a period of 75 minutes. Peptides were collected in a 96-well plate over a 65 min-gradient of 13–44%B with Buffer A comprising 5% acetonitrile, 10 mM ammonium bicarbonate, pH 8 and Buffer B comprising 90% acetonitrile,10 mM ammonium bicarbonate, pH 8. Fractions were then pooled into 24 samples, followed by sample clean-up using the Stage Tip protocol. This protocol uses C18 Empore^TM^ Extraction Disks (Fisher Scientific, Catalog Number 14-386-2). The matrix was primed with methanol and equilibrated with 70% acetonitrile, 1% FA followed by washing twice with 1% FA, loading the sample in 1% FA, followed once again by two 1% FA washes, and finally peptide elution using 70% acetonitrile, 1% FA. Samples were dried before resuspension in MS Loading Buffer (3% acetonitrile, 5% FA).

#### LC-MS

Peptides were injected onto a 30 cm, 100 μm (internal diameter) column and separated using an EASY-nLC 1000 HPLC (ThermoFisher, Catalog Number LC120). The flow rate was 450 nl/min with a gradient of 6–28%B over 170 minutes with Buffer A comprising 3% acetonitrile, 0.4% FA and Buffer B comprising 100% acetonitrile, 0.4% FA. The column was packed with 1.8 μm C18 beads with a pore size of 12 nm (Sepax Technologies Inc.) heated to 60 °C using a column heater (constructed in-house). Samples from the HPLC were injected into an Orbitrap Fusion Lumos Tribrid MS (ThermoFisher, Catalog Number FSN02-10000) using a multi-notch MS3 method^17,18^. MS scans were performed in the Orbitrap over a scan range of 400–1400 m/z with dynamic exclusion. The top 10 ions with charge states from 2 to 6 were selected for MS/MS. Rapid rate scans were performed in the Ion Trap with a collision energy of 35% and a maximum injection time of 120 ms. TMT quantification was performed using SPS-MS3 in the Orbitrap with a scan range of 100–1000 m/z and an HCD collision energy of 55%. Orbitrap resolution was 50,000 (dimensionless units) with a maximum injection time of 120 ms. MS isolation windows were varied depending on the charge state.

### Phosphopeptide Enrichment and Mass Spectrometry

Following protein digestion as described above, approximately 1 mg of protein was removed from samples corresponding to each differentiation time point and then desalted and purified using SepPak tC18 Vac RC Cartridges. Phosphopeptides were enriched using Titansphere 5 μm titanium dioxide beads (GL Sciences Inc.) prewashed with 50% acetonitrile/2M lactic acid. Phosphopeptides were resuspended in 50% acetonitrile/2M lactic acid and incubated with beads for 1 hour, followed by washing successively with 50% acetonitrile, 0.1% trifluoroacetic acid (TFA) and 25% acetonitrile, 0.1% TFA and then eluted twice with 50 mM potassium phosphate, dibasic, pH 10. Phosphopeptides were then subjected to TMT labelling after which the samples were combined and purified using SepPak cartridges as described above. Phosphotyrosine-containing peptides were enriched using a p-Tyr-1000 rabbit antibody (Cell Signaling Technology, P-Tyr-1000, Catalog Number 8954). The antibody was coupled to protein A-agarose beads (Sigma-Roche, Catalog Number 11719408001) by incubation overnight at 4 °C and washed with cold PBS and the antibody-bead complex was then incubated with TMT-labelled phosphopeptides for 3 hours at 4 °C. Phosphotyrosine peptides were eluted twice with 0.15% FA, followed by Stage Tip desalting and LC-MS as described above. HPLC separation of the eluate was performed as previously described prior to LC-MS^19^.

### Immunofluorescence Procedures

ReNcell VM cells were plated on Matrigel-coated Nunc™ MicroWell™ 96-Well Optical-Bottom Plates (ThermoFisher, Catalog Number 165305) at a density of 10,000 cells/well. Undifferentiated (UD) and differentiated cells (Day 5 and Day 15) were fixed in 4% paraformaldehyde (PFA in PBS pH 7.4, freshly diluted from a 16% stock purchased from Electron Microscopy Services, Catalog Number EMS 15710) at room temperature for 30 minutes and washed with PBS four times. Cells were permeabilized with ice-cold methanol for 10 minutes at room temperature followed by four washes in PBS. Cells were blocked by incubation in Odyssey blocking buffer (LI-COR Biosciences, Catalog Number 927-40001) at room temperature for 1 hour. Unconjugated primary antibodies were diluted in Odyssey blocking buffer and added to fixed cells at 4 °C for 12 hours. Antibody identities, species and dilutions are shown in Synapse (syn11701219, Data Citation 1). Plates were washed with PBS four times and incubated at room temperature for 1 hour with fluorescent secondary antibodies diluted 1:400 in PBS. To stain nuclei, cells were incubated at room temperature for 15 minutes with 0.2 μg/ml Hoechst 33342 (Invitrogen, Catalog Number H3570) and washed with PBS. For fluorescently-conjugated primary antibodies, incubation with secondary antibodies was omitted. Samples collected at different time points after the initiation of differentiation were treated with the same batch of diluted antibodies and processed at the same time to facilitate inter-sample comparison.

### Fixed Cell Microscopy and Image Processing

Images were acquired at room temperature using a plate-based laser confocal microscope, the InCell Analyzer 6000 (GE Healthcare), with a Nikon 20X, 0.45NA objective, four lasers (Ex405nm, Ex488nm, Ex561nm and Ex640nm) and a cooled-CMOS camera. The pinhole size was set to 1.2 AU to promote confocality. Exposure times were 0.1 second for the Ex405 laser and 0.4 second for the remaining lasers. Laser auto-focus was used to adjust the focal plane for each well, after which six fields of view were acquired per well at locations selected at random across the well. No binning was used and Z stacks were not acquired. A minimal amount of post-acquisition processing was applied to images using IN Cell Analyzer software (GE Healthcare Life Sciences) primarily to adjust brightness and contrast. The same settings were used on all images prior to export for analysis using IN Cell Analyzer software. Image processing and data interpretation were performed with the investigator blinded to sample identity.

### Live-cell Imaging and Data Processing

ReNcell VM cells were plated and differentiated on 96 well plates as described above. Single-cell tracking is challenging in these cultures because high cell density is required for viability. To overcome this problem, GFP-expressing ReN-G cells were mixed with non-GFP expressing cells at a ratio of 1:50. An IncuCyte microscope (Essen Bioscience) captured images directly from multi-well plates inside a tissue culture chamber, allowing for extensive live cell imaging over a 15 day period without unduly perturbing samples. Imaging was performed with a 10X objective (generating a 1392 × 1040 pixel image of an entire multi-well plate at a nominal resolution of 1.22 μm/pixel) using either phase contrast for unlabelled cells or fluorescent mode and a GFP-selective filter for ReN-G cells. The acquisition interval was set to 3 hours and exposure time to 0.4 second. For each time point, four fields were exported as .tiff files without any additional software-based manipulation; one representative .tiff image from each time point is available for download (syn11691420, Data Citation 1). Cell confluence calculations were based on the sum of cell bodies and processes detected and performed using IncuCyte Analyzer Software (Essen Bioscience) provided with the instrument.

### Data Analysis

A compilation of commercially available software (Core software program described in the Code Availability section) was used to convert mass spectrometric data (Thermo “.RAW” files) to mzXML format and to correct monoisotopic m/z measurements and erroneous peptide charge state assignments. Assignment of MS/MS spectra was performed using the Sequest^20^ (version 28^21^ (http://fields.scripps.edu/yates/wp/?page_id=17)) and the Human UniProt database (downloaded February 2014). The database search included reversed protein sequences and known contaminants such as human keratins which were excluded for subsequent analyses. Linear discriminant analysis was used to distinguish forward and reverse hits^22^. Peptides were identified using an MS2 spectrum and a false discovery rate (FDR) <1% was achieved by applying the target-decoy database search strategy^22^. Filtering was performed as described previously^18^. Variable extents of modification including the presence of oxidized methionine and over-labelling of TMT on serine, threonine and tyrosine^23^ were considered during peptide assignment for whole-protein experiments. Methionine oxidation and phosphorylation on serine, threonine and tyrosine were used as variable modifications for phosphoproteomics experiments. For protein identification and quantification, shared peptides were collapsed into the minimally sufficient number of proteins using rules of parsimony. Peptides with a total TMT value of >200 and an isolation specificity of >0.7 were included for quantification. For phosphopeptides, site localization confidence was assessed using the A-score method^24^, and a score above 13 indicated good confidence in phosphosite localization^25^.

### Code Availability

PCA, hierarchical and k-means clustering, differential expression and enrichment analyses were performed using code written in Python (https://github.com/datarail/msda). The optimal number of clusters (k) for k-means clustering was determined by computing silhouette scores. The scores were comparable, ranging from 0.21 to 0.25 for k = 5 to k = 11 respectively. We chose k = 8 since the resultant clusters could be distinguished qualitatively based on differences in temporal dynamics. The neuronal gene set library (syn12578559, Data Citation 1) was customized and downloaded from GSEA by searching for terms across all libraries that contained “neuron” in their name. All code that takes datasets from Synapse and computes and plots the results shown in Figs 2–5 is available at https://github.com/labsyspharm/rencell. Analysis of live-cell microscopy data was performed using Incucyte Zoom software (Version 2016B, Essen BioScience) which is provided with the instrument.

## Data Records

This paper describes nine datasets of two different types: (i) TMT LC-MS of total protein or phosphopeptides acquired at ten time points over a 15-day period following differentiation of ReNcell VM in the absence of growth factors (syn11691421, Data Citation 1), and (ii) images of cells undergoing the same differentiation process and acquired using either live-cell or immunofluorescence microscopy (syn11691420, Data Citation 1). All data are publicly available for download via Sage Bionetworks Synapse. Metadata, including protocols and lookup tables are also provided in this repository (syn11667815, Data Citation 1). In addition, all primary proteomic datasets are also available at ProteomeXchange via the PRIDE archive (Data Citation 2).

### Total Proteomics

Baseline proteomics data (Fig. 2) (syn11701290, Data Citation 1) comprises TMT LC-MS data on protein abundance over the course of ReNcell VM differentiation; biological replicates were analysed for each time point. Data is reported as the number of peptides quantified in both replicates, and as normalized, scaled and summed TMT signal/noise values. Proteins were grouped into several K-means clusters as indicated (syn11701290, Data Citation 1, see column named “kmeans_cluster_name”) and shown in Fig. 2c. Experimental and analytical methods and results are also available at http://lincs.hms.harvard.edu/db/datasets/20345/.

### Phosphoproteomics

Baseline phosphoproteomics data (Fig. 3) (syn11701291, Data Citation 1) involves phosphopeptide enrichment TMT LC-MS performed on the same samples as in the total proteomics. Syn11701291 consists of the exported phosphopeptide list from Core software processed as described above. Data are presented as normalized, scaled, summed TMT signal/noise values of the phosphopeptides. This table also indicates which K-means cluster each phosphopeptide fell into (Fig. 3c). Experimental and analytical methods and results are also available at http://lincs.hms.harvard.edu/db/datasets/20346/.

Drug perturbation datasets (Fig. 4) (syn12560131, Data Citation 1) report TMT LC-MS data at ten timepoints for cells treated with small molecule drugs previously reported to impact ReNcell VM differentiation. These include 2 μM kenpaullone (syn11701292, Data Citation 1), 2 μM mevastatin (syn11701293, Data Citation 1)) or a DMSO-only control (syn11701294, Data Citation 1). Data are reported as number of peptides quantified in singlicate, and as normalized, scaled, summed TMT signal/noise values. Experimental and analytical methods and results are also available at http://lincs.hms.harvard.edu/db/datasets/20347/.

### Imaging

Microscopy datasets (syn11691420, Data Citation 1) comprise representative imaging data collected at 3-hour intervals over 15 days of ReNcell VM differentiation. For each condition, one .mp4 movie and 121.tiff files derived from the live-cell data were recorded. These represent standard conditions equivalent to those used for baseline mass spectrometry (Fig. 5a–c) (syn11725079, Data Citation 1), as well as cells treated with a DMSO vehicle-only control (Fig. 10) (syn11725208, Data Citation 1), cells treated with 2 μM kenpaullone (Fig. 10) (syn11725209, Data Citation 1) and cells treated with 2 μM mevastatin (Fig. 10) (syn11725210, Data Citation 1). Immunofluorescence imaging of cell markers is presented in Figs. 6–9. Confluence was calculated based on the sum of cell bodies and processes detected over the course of a 7-day differentiation period in cells treated with a DMSO vehicle-only control or with 2 μM kenpaullone (syn11701318, Data Citation 1).

### Metadata

See “Data_descriptor_worksheet.xlsx” (syn17023698, Data Citation 1) for a complete list of samples and assays employed in this study. In addition, our data repository contains all related protocols, dataset tables and figures. Refer to Synapse (syn17052375, Data Citation 1) for metadata on instrument specification, experimental design and analyses pertaining to mass spectrometry-based quantification as specified by MIAPE standards (http://www.psidev.info/node/91).

## Technical Validation

### Reproducibility of Differentiation with Passage Number

ReNcell VM have been reported to retain normal diploid karyotype over 45 passages in culture (http://www.emdmillipore.com/US/en/product/ReNcell-VM-Human-Neural-Progenitor-Cell-LIne,MM_NF-SCC008). All data in this paper were obtained from cells that had been propagated for fewer than 15 passages. To further examine the reproducibility of ReNcell VM differentiation, we used an IncuCyte live-cell microscope to compare differentiation time courses of cells passaged 3 to 10 times. Separate cultures were initiated from two different frozen vials (these represent biological duplicates); two wells per biological duplicate were examined up to 7 days for each culture at passages 3, 4, 5, 8 and 10 (P3, P4, P5, P8 or P10) (Fig. 5d) (syn11701318, Data Citation 1). We found that the pattern of differentiation under standard conditions was reproducible as measured by the increase in neuron/glia confluency and neurite outgrowth. We also found that kenpaullone significantly and reproducibly promoted neurite outgrowth (as previously reported^14^) and increased cell confluency as compared with the DMSO control. These differences were consistent among various passages, demonstrating that ReNcell VM cells can be used reliably to study patterns of human neuronal cell differentiation under normal and drug-perturbed conditions.

### Mass Spectrometry Quality Control

Quality control checks for mass spectrometry were incorporated at multiple points in the workflow. To test for efficient digestion of samples, defined as <15% of potential sites of proteolysis left uncleaved, a “Digest Check” was performed using LC-MS/MS as described in the methods section. TMT labelling efficiency aims for modification of >98% of available sites and was confirmed by LC-MS analysis. A “Ratio Check” was performed using LC-MS3 to determine relative amounts of labelled peptides in each of the 10-plex samples, as described in the methods section.

## Usage Notes

### Characterization of morphological changes during ReNcell VM differentiation

Significant phenotypic and morphological changes take place during neural differentiation. To characterize these changes in ReNcell VM cultures, time-lapse microscopy was performed over a 15-day period. These data confirmed sample-to-sample consistency in ReNcell differentiation in the absence of drugs and the previously reported ability of kenpaullone to accelerate development (Fig. 5d) (syn11701318, Data Citation 1). Immunofluorescence (IF) staining at 0, 5 and 15 days using lineage-specific markers (beta III tubulin (Tubb3) and NeuN for neurons; Glial Fibrillary Acidic Protein (GFAP) for astrocytes; and Proteolipid Protein 1 (PLP1) for oligodendrocytes) also confirmed previous data showing that ReNcell VM progenitor differentiate into a mixed culture of neurons, astrocytes and oligodendrocytes (Figs 6 and 7). Markers of proliferation fell substantially within 3 days of growth factor withdrawal, as evidenced by a >10-fold reduction in the levels of Ki67, a marker of cell proliferation (Fig. 10). Conversely, neuronal and glial-specific markers increased over the course of differentiation; this included a neuron-specific tubulin isoform (Tubb3) and microtubule-associated proteins (Figs 6, 8, 9). The levels of GFAP, which selectively labels astrocytes, and PLP1, which labels oligodendrocytes, also increased during differentiation (Fig. 6). Consistent with previous reports that differentiated ReNcells are neurophysiologically active we observed upregulation of markers of axon maturation (Microtubule Associated Protein Tau, MAPT), dendritic development (Microtubule Associated Protein 2, MAP2), and synaptogenesis (Synaptophysin, SYP) (Figs 7, 8, 9). Overall, changes in immunostaining were congruent with proteomic data (Figs 6–10).

Live-cell imaging studies performed on cell cultures sparsely seeded with labelled ReN-G neurons (to enable single-cell tracking, Fig. 10) made it possible to follow neurite outgrowth. Both mevastatin and kenpaullone accelerated outgrowth as compared to a DMSO-only control, with the most dramatic changes being increased neurite length and branching by 3 days that lasted through day 7. This correlated well with proteomic data showing that mevastatin and kenpaullone induced significant changes in specific protein levels as early as 6 hours after the initiation of differentiation (e.g. HMGCR in mevastatin treated cells and GSK3 α and β in kenpaullone treated cells; Fig. 4) (syn12560131, Data Citation 1).

### Molecular pathways associated with ReNcell VM differentiation

Neural differentiation is a complex process involving changes in protein expression, translocation and modification. K-means clustering of proteomic data yielded multiple protein clusters with distinct temporal trajectories. We observed (i) three clusters in which protein abundance increased at either early, middle or late times, (ii) two clusters in which abundance decreased early or late and (iii) two clusters in which abundance increased transiently and then fell again (Fig. 2c) (syn11701290, Data Citation 1). Differentiation-dependent decreases in the levels of cell cycle proteins (e.g. cyclin-dependent kinases, CDKs^26^) were observed (downregulated cluster in Fig. 2c). Enrichment analyses using a custom neuronal gene set library also revealed changes in *shh* pathway components^27^; protein clusters that were downregulated upon differentiation included *shh* proteins found in neural progenitor cells from the ventral mesencephalon region of the human foetal brain (Figure 2d). Within this set of downregulated proteins, different CDKs were differentially regulated. For example, levels of CDK1, 2, 4 and 6 dropped quickly by day 2 of differentiation while CDK7, 8, 9, 12 and 13 gradually decreased until day 14. CDK5 was an exception in that its level was observed to increase as neurons started to differentiate and form neurites, consistent with its proposed role in neurite extension and arborisation^28^.

Many proteins involved in axon guidance and neurite outgrowth increased transiently early during differentiation (days 1-3) and then plateaued (see Fig. 2c ‘upregulated mid-to-late stage’ cluster): e.g. Semaphorin 5B (SEMA5B), Neuroligin 1 and 2 (NLGN1 and 2) and Doublecortin (DCX). Others increased steadily from day 2 to 15 (see Fig. 2c ‘upregulated’ cluster): e.g. Plexin B1(PLXNB1), Neuroligin 3 (NLGN3) and Roundabout Guidance Receptor 2 (ROBO2). In contrast, synaptic proteins, neurotransmitters, neurotransporters and modulators of synaptic transmission (e.g. Synaptophysin (SYP), Microtubule Associated Protein Tau (MAPT), Solute Carrier Family (SLC1A2, SLC6A11 and SLC6A1)) were upregulated primarily at later time-points (days 7–15, see Fig. 2c ‘upregulated late’ cluster), consistent with a role in formation of functional neural networks in mature neurons. Brain developmental proteins (e.g. RE1 Silencing Transcription Factor (REST corepressors), exosome complex components, and Polypyrimidine Tract Binding Protein 2 (PTBP2)) gradually decreased as neurons matured (Fig. 2c ‘downregulated mid-stage’ cluster).

Reproducible changes in phosphorylation states were also observed over the course of ReNcell VM cell differentiation (Fig. 3a,b). As with total protein data, phosphoproteins clustered by day of differentiation into three distinct groups: days 0 to 1, days 2 to 4 and days 7 to 15. In addition, k-means clustering grouped peptides based on distinct temporal profiles over the differentiation process. Substantial changes were observed in the phosphorylation of cytoskeletal proteins (e.g. vimentin and stathmin), cell cycle markers (e.g. Checkpoint Kinase 1 (CHEK1), adhesion molecules (e.g. Cadherin) and signalling pathways (e.g. CDK, Wnt, Mechanistic Target of Rapamycin Kinase (mTOR), Mitogen-activated Protein Kinases (MAPK), mTOR, MAPK, etc.) (Fig. 3c), some of which have been previously reported, including cell cycle regulators^26^, GSK3β signalling^29,30^, Wnt/β-catenin signalling^31,32^, mTOR pathway^33,34^ and MAPK cascades^35,36^; changes in the phosphorylation of cytoskeletal proteins were also observed (e.g. vimentin^37,38^ and stathmin^39,40^) (syn11701291, Data Citation 1). Using enrichment analyses against the custom neuronal gene set library, we were able to identify changes in pathways associated with initiation of neural differentiation, axonal and dendritic development, synaptic formation and neuronal maturation (Fig. 3d).

### Pathways altered by drugs that promote ReNcell VM differentiation

There is considerable interest in pharmacological agents that induce differentiation of stems cells in culture as a potential means to rescue neurodevelopmental abnormalities. We used total shotgun proteomics and imaging to study the effects of two drugs, kenpaullone and mevastatin, previously reported to promote differentiation of ReNcell-VM cultures^14^. Protein abundance for a given treatment at each day was normalized to the level of protein at day 0 under the same treatment condition and then log-transformed (yielding data on log2 fold change). Based on PCA (Fig. 4a), differences in the duration of differentiation projected primarily along PC1 (60% of variance explained), while differences between treatments were observed along PC2 (15% of variance explained). Proteomic data from drug-treated cells did not include replicates and we therefore improved our ability to identify differentially regulated proteins by averaging, for each drug, the four samples collected between days 7 and 15; PCA and hierarchical clustering showed that changes in protein abundance over this period were modest.

In cells exposed to kenpaullone (a pan-specific GSK3 and CDK inhibitor), neuronal development markers such as ROBO3 which is involved in axon guidance, and DCX and TUBB3 which are cytoskeletal proteins biased towards neuronal lineage exhibited a significantly greater increase in abundance (Fig. 4b and d). Kenpaullone treatment slightly reduced the total protein levels of GSK3α and β as well as reducing CDK1 and CDK12 at later time points without affecting CDK6. Further experiments using phosphoproteomics may shed light on specific signalling pathways critical for neural differentiation.

In the case of mevastatin treatment, its target HMG-CoA reductase, a rate-limiting enzyme in biosynthesis of cholesterol and nonsterol isoprenoids, increased significantly over the course of differentiation while the level dropped in both DMSO control- and kenpaullone-treated samples. Since cholesterol plays a role in the *shh* pathway and in the synthesis of steroids^41,42^, such an increase may explain why mevastatin favors maturation of dopaminergic neurons in the ReNcell VM line^14^. Our data are also consistent with the notion that protein prenylation (which is inhibited by statins) may act as a brake on axonal growth^43^. Further experiments focused on the dynamics of gene expression, protein modification, localization and function of HMG-CoA reductase/mevalonate pathway therefore seem warranted. Interestingly, mevastatin treatment also caused a large decrease in the level of CyclinA2 (CCNA2), a marker for cell proliferation, which did not change during differentiation under DMSO. The reduction occurred within the first day of differentiation and was sustained throughout the time course (Fig. 4c,d). The molecular mechanisms by which statins promote neural differentiation is largely unknown. One possibility is that statins activate Epidermal Growth Factor Receptor (EGFR), Extracellular Signal-regulated Kinases (ERK1/2) and AKT/Protein Kinase B (PKB)^44^ signalling; we observed changes consistent with a mechanism during ReNcell VM differentiation.

Immunofluorescence and proteomic data showed that differentiation-induced changes in protein levels were comparable in many cases in the presence and absence of drugs (e.g. CDK6, MAPT, GFAP, PLP1, etc.). Particularly in the case of mevastatin, the final proportion and identity of neuronal and glial cells appeared to be similar; in contrast, by day 15, kenpaullone-treated cells differed from control cells along PC2. Treatment with either mevastatin or kenpaullone shifted some changes in protein expression forward in time; for example, markers of cell proliferation such as CCNA2 were downregulated sooner than in control cells while markers for neuronal development and migration such as Netrin 1 (NTN1) were upregulated sooner (see Fig. 4d) (syn12560131, Data Citation 1). This is consistent with live imaging data showing earlier neurite outgrowth as well as enhanced neurite length and branching (Fig. 10).

### Unique usage of the multidisciplinary datasets

Ways to analyse proteomics data are reviewed elsewhere^45,46^. The data in this paper are unusual in combining mass spectrometry with fixed and live-imaging as a means to better characterize differentiation of ReNcell VM cell^7^ under both standard conditions of growth factor withdrawal and in the presence of mevastatin (an HMG-CoA reductase inhibitor) and kenpaullone (a poly-selective CDK and GSK3 inhibitor).

Altogether, this study supports the utility of the ReNcell VM culture model as a means to study the differentiation of immortalized precursor cells into neurons, astrocytes and oligodendrocytes. Differentiation time-courses are reproducible across biological repeats and the previously reported ability of mevastatin and kenpaullone to accelerate differentiation is readily apparent at the morphological and proteomic level. Using the protein profiling data we have collected, follow-up experiments using immunofluorescence or focused proteomics could be readily designed to study a wider range of genetic and small molecule perturbagens. The primary limitation in the data reported here is that bulk proteomics does not distinguish among neurons, astrocytes and oligodendrocytes; however, this could be addressed in the future by more extensive use of imaging and single cell profiling.

## Additional Information

**How to cite this article**: Song, Y. *et al*. A dynamic view of the proteomic landscape during differentiation of ReNcell VM, a human neural progenitor line. *Sci. Data*. 6:190016 https://doi.org/10.1038/sdata.2019.16 (2019).

**Publisher’s note**: Springer Nature remains neutral with regard to jurisdictional claims in published maps and institutional affiliations.

## Supplementary Material
